# Lectins as Natural Antibiofilm Agents in the Fight Against Antibiotic Resistance: A Review

**DOI:** 10.3390/molecules30163395

**Published:** 2025-08-15

**Authors:** Thiago Henrique Napoleão, Thiago Lucas da Silva Lira, Emmanuel Viana Pontual, Gustavo Ramos Salles Ferreira, Pollyanna Michelle da Silva

**Affiliations:** 1 Departamento de Bioquímica, Centro de Biociências, Universidade Federal de Pernambuco, Recife 50670-901, PE, Brazil; thiago.silvalira@ufpe.br (T.L.d.S.L.); gustavo.rsferreira@gmail.com (G.R.S.F.); pollyanna.msilva@ufpe.br (P.M.d.S.); 2 Departamento de Morfologia e Fisiologia Animal, Universidade Federal Rural de Pernambuco, Recife 52171-030, PE, Brazil; emmanuel.pontual@ufrpe.br

**Keywords:** biofilm inhibition, biofilm eradication, antibiotic tolerance, antimicrobial proteins

## Abstract

Biofilms are complex microbial communities embedded in a self-produced extracellular polymeric matrix. These structures confer increased resistance/tolerance to antimicrobial agents and immune responses, posing a serious challenge in both clinical and industrial contexts. In response to these challenges, increasing attention has been given to the development of novel antibiofilm strategies. Among the promising alternatives are lectins—carbohydrate-binding proteins. This review explores the structural and functional features of biofilms and critically discusses recent studies reporting the antibiofilm effects of lectins. Additionally, it addresses the main challenges and limitations surrounding the practical application of lectins to combat biofilms. Lectins from plants, animals, and microorganisms have shown potential to inhibit biofilm formation by disrupting the extracellular matrix, modulating quorum sensing, and affecting bacterial motility and metabolism. Additionally, they can eradicate established biofilms by degrading the matrix, killing or removing microbial cells, and/or preventing biofilm reformation. Together, the findings reviewed here support the continued investigation of lectins as potential agents against biofilm-associated infections as well as highlight the need to address existing gaps, such as the lack of in vivo studies and limited research on the structure–function relationships of lectins and their antibiofilm activity.

## 1. Introduction

Microorganisms represent the greatest biodiversity on Earth and play a fundamental role at the base of the food chain in all ecosystems of the biosphere. They exist as single cells or within colonies and can live in symbiosis with multicellular organisms, including humans. Microorganisms also possess significant biotechnological potential and are widely used in industry; for example, they can act as biocatalysts capable of producing natural compounds such as flavors, biosurfactants, polysaccharides, and oils [1,2]. However, many microorganisms are also responsible for diseases in humans, animals, and plants. Estimates indicate that 92.0 million deaths (range: 82.8–102.0 million) caused by microbial infections could cumulatively occur between 2025 and 2050 [3].

Considering such threats, the discovery of antimicrobial drugs represented a revolutionary milestone in the fight against infectious diseases. Nevertheless, microbial resistance has posed a challenge to infection control since its inception, primarily due to the intense selective pressure imposed by antimicrobial use [4]. Thus, multidrug-resistant strains have rapidly spread in hospitals worldwide. For example, in the 1980s, third-generation cephalosporins were introduced to combat *Enterobacteriaceae* resistant to existing β-lactam antibiotics. However, the extensive and often inappropriate use of β-lactam antibiotics in both clinical and agricultural settings has driven the emergence and dissemination of bacterial isolates that produce extended-spectrum β-lactamases, enzymes that degrade nearly all β-lactam antibiotics [5].

While resistance enables bacteria to survive antimicrobial treatment, successful infection depends on an array of virulence strategies that allow pathogens to colonize the host and cause disease. The infection steps include transmission, colonization, adhesion, and invasion. Common adhesion mechanisms include fimbriae and adhesins, while invasion is mediated by invasins that bind to host cell receptors. Interestingly, some Gram-positive bacteria, such as *Clostridium tetani* and *Corynebacterium diphtheriae*, do not need to adhere to host cells to exert their virulence. Instead, they release toxins encoded by virulence genes that damage host tissues [6]. Bacterial pathogens produce a wide variety of virulence factors to facilitate colonization and host damage. These include toxins, modulins, aggressins, capsules, pigments, enzymes, and biofilms. The expression of virulence genes is regulated by the quorum sensing (QS) system, which responds to population density through the detection of small signaling molecules [7].

Among the various virulence factors, the ability to form biofilms represents a crucial survival strategy, critically enhancing bacterial persistence and resistance to antimicrobial agents [7]. Microorganisms form biofilms when they assume the sessile (surface-attached) form rather than the planktonic state (free-floating). Between 65% and 80% of infections are associated with biofilm-forming bacteria, and biofilm development is tightly regulated by the QS system [8]. Microorganisms within biofilms can tolerate antibiotic concentrations thousands of times higher than those needed to inhibit planktonic cells. This increased tolerance results from multiple factors: antibiotics may bind to the extracellular polymeric matrix (EPM), limiting their diffusion and effectiveness in a process known as antibiotic sequestration; extracellular enzymes within the biofilm can degrade antibiotics; efflux pumps, often regulated by QS, actively expel antibiotics from bacterial cells; high biofilm cell density reduces drug efficacy; horizontal gene transfer—including transformation, transduction, and conjugation—occurs more frequently in biofilms, promoting the spread of resistance genes; and cells within biofilms often exhibit reduced metabolic activity (see Section 2), further enhancing tolerance [8,9,10,11,12,13,14].

In the current scenario of increasing antimicrobial resistance, there has been a heightened search for compounds capable of inhibiting biofilm formation by microorganisms, as well as disrupting biofilms that have already formed. This review addresses the potential of lectins (carbohydrate-binding proteins) as antibiofilm agents against bacteria, highlighting the current challenges and limitations related to their effective application. We first discuss the key features of bacterial biofilms and provide an overview of lectins, followed by a comprehensive review of studies reporting the antibiofilm activity of these proteins.

## 2. Biofilms

Biofilms are heterogeneous biological communities characterized by a high degree of structural organization. They consist of microbial cells embedded within a self-produced EPM, which includes exogenous DNA (eDNA), proteins, amyloids, and lipids. This three-dimensional structure enables biofilms to adhere to both living and non-living surfaces [14,15,16,17]. The EPM protects the microbial community from environmental stressors such as ultraviolet radiation and host immune responses, while also facilitating the diffusion of water and nutrients and promoting genetic exchange among cells [9,14].

**Figure 1:**
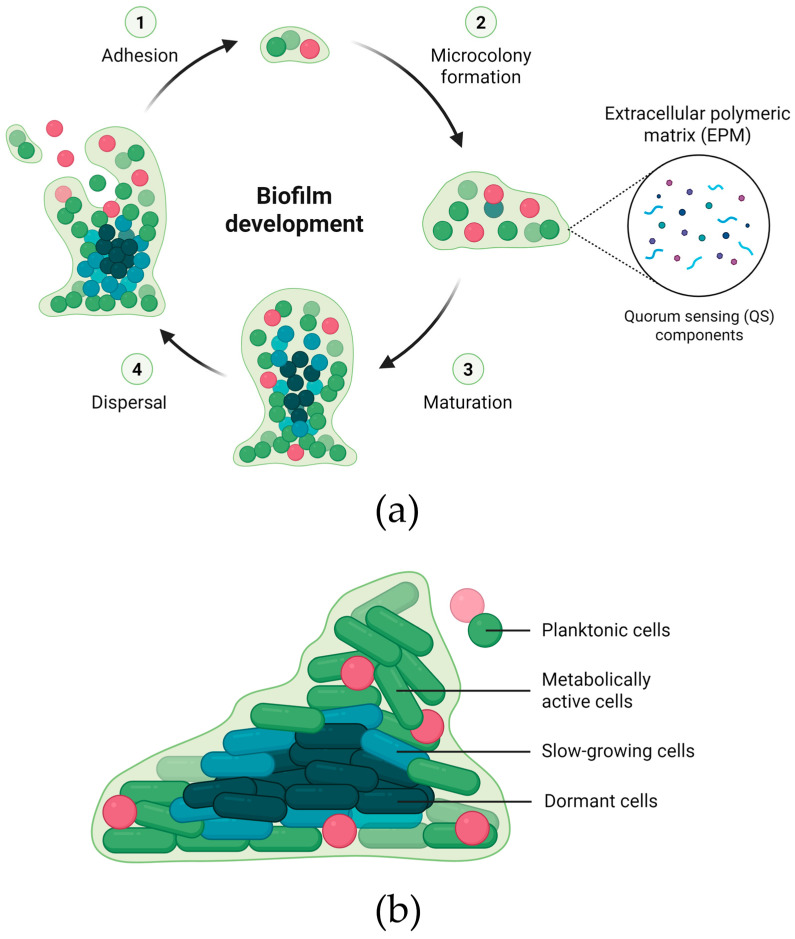
Formation and structural organization of microbial biofilms. (**a**) Schematic representation of the four main stages of biofilm development: (1) initial adhesion of planktonic cells to a surface; (2) microcolony formation, driven by cell proliferation and the onset of chemical communication; (3) maturation, characterized by the accumulation of an extracellular polymeric matrix (EPM) that stabilizes and protects the biofilm; and (4) dispersal, during which cells detach and return to the planktonic state to colonize new niches. These processes are regulated by quorum sensing (QS) system, through signaling molecules released for intercellular communication. (**b**) Cross-sectional view of a mature biofilm, illustrating its heterogeneous structure. Planktonic cells are free-living microbial forms capable of detaching from mature biofilms to seed new sites of colonization. The outer layers are composed predominantly of metabolically active cells responsible for growth and defense. In contrast, the inner regions harbor slow-growing and dormant cells with extremely low metabolic activity, which exhibit high tolerance to antibiotics and environmental stress, contributing to the resistance and persistence of microbial biofilms.

The development of a biofilm is a dynamic and highly regulated process that occurs in distinct stages. It begins with the initial adhesion of cells to a surface via flagella or physicochemical interactions (e.g., Van der Waals forces or electrostatic attraction), followed by microcolony formation through cell proliferation and chemical signaling. As the biofilm matures, cells produce the EPM, establishing a complex and resilient structure. In the final stage, known as dispersal, cells multiply and detach, returning to the planktonic state [16,18]. Matrix proteins act as adhesins that promote the aggregation of planktonic cells and stabilize the structural integrity of the community [14]. Biofilm formation is coordinated by QS components, involving secondary messengers, signaling molecules, and small RNAs (Figure 1a) [17].

In addition to living microbial cells, biofilms often contain spores, dead or lysed cells which release eDNA and cellular debris, and even host immune cells such as neutrophils and macrophages, which can become trapped within the biofilm. The microbial community (primary inhabitants) may include bacteria, fungi, algae, cyanobacteria, and archaea. Biofilms frequently consist of two or more microbial species, whose interactions contribute to structural and functional properties not observed in single-species biofilms [19]. They can also harbor microbial cells in various growth phases and ages (Figure 1b). The outer layers of the biofilm are predominantly composed of metabolically active cells, whereas slow-growing or metabolically inactive persister cells are typically located in the deeper regions. These cells, often referred to as dormant cells, are capable of surviving antibiotic treatment and harsh conditions, thereby contributing to the resilience of biofilm resilience and the persistence of chronic infections [13].

Another key feature of biofilms is the spatial heterogeneity that arises from gradients of cells and molecules within the community. Extracellular enzymes in the matrix create a digestive system that allows the utilization of sequestered or accumulated nutrients and supports the recycling of biopolymers, eDNA, and cellular debris, as previously described [17,20].

Biofilms are typically stationary structures, but some movement can occur within them. Type IV pili (surface structures composed of pilin proteins found in Gram-negative and some Gram-positive bacteria) are involved in various forms of motility. These include the movement of large microcolonies across surfaces through merging, social motility for bacterial predation, and the development of complex structures such as fruiting bodies. Collective bacterial movement may also involve gliding motility of microcolonies, which facilitates bacterial cargo transport and microbial adaptation [21,22].

These structured communities are implicated in numerous human diseases, including prostatitis, dental caries, rhinosinusitis, otitis, cystic fibrosis, endocarditis, osteomyelitis, lung infections, and urinary tract infections [23,24]. For example, biofilms formed by *Enterococcus* spp. are frequently found in wounds, urinary and gastrointestinal tract infections, and cases of endocarditis [25]. Biofilms also commonly develop on medical devices such as catheters, orthopedic implants, contact lenses, and implantable electronic devices, contributing to high rates of device-associated infections [23,26]. The microbial composition of a biofilm depends on the type of device and the duration of its use. For instance, the bacterial profile on central venous catheters is influenced by the fluid inside the catheter: Gram-positive bacteria such as *Staphylococcus epidermidis* and *Staphylococcus aureus* grow poorly in certain intravenous fluids, whereas Gram-negative bacteria like *Pseudomonas aeruginosa* thrive in these environments [16]. Le et al. [27] demonstrated that biofilms formed by *S. epidermidis* can evade the host’s innate immune system and persist on implanted surfaces, making early diagnosis challenging and treatment more complex in advanced stages.

Multispecies biofilms are commonly found on medical devices and are responsible for a significant number of associated infections, posing a serious threat to public health and healthcare systems [28,29]. For example, multispecies biofilms involving uropathogenic bacteria such as *Proteus* spp., *Staphylococcus* spp., *Providencia* spp., and *Ureaplasma* spp. are frequently associated with catheter-associated urinary tract infections and the formation of infectious urinary stones [30].

## 3. The Antibiofilm Activities of Lectins

**Figure 2:**
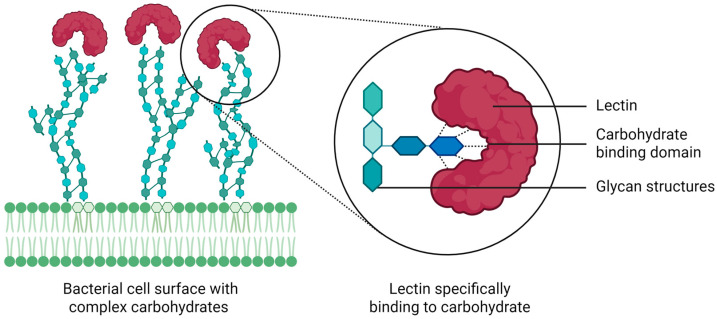
Carbohydrate-binding capacity of lectins. Lectins are characterized by their ability to selectively recognize and bind complex carbohydrate structures, such as oligo- and polysaccharides. The figure illustrates the interaction between a lectin and a bacterial cell surface displaying complex carbohydrates. The zoom-in highlights the lectin’s carbohydrate binding domain interacting with specific sugar residues. This binding is reversible and occurs without chemical modification of the target. The ability of lectins to recognize glycans with high specificity underlies a wide range of biological activities, including antimicrobial and antibiofilm effects.

Lectins are carbohydrate-binding proteins widely found in microorganisms, plants, and animals. In plants, lectins can be isolated from a variety of tissues, including bark, roots, leaves, flowers, seeds, and fruits. They contain at least one non-catalytic domain capable of reversibly binding to mono-, oligo-, or polysaccharides without chemically modifying the carbohydrate moiety (Figure 2) [31,32].

Notably, lectins typically exhibit a higher affinity for complex glycans than for simple mono- or oligosaccharides [31,32]. Owing to their specific carbohydrate-binding capabilities, lectins display a broad spectrum of biological activities, such as immunomodulatory [33], antihelmintic [34], antiviral [35], insecticidal [36], anticancer [37], anti-inflammatory [38] and antimicrobial effects. Table 1 provides an overview of the lectins discussed in the following sub-sections regarding their potential as antibiofilm agents against bacteria.

### 3.1. Inhibitory Effect of Lectins on Biofilm Formation

Inhibition of biofilm formation refers to the process of preventing or limiting the development of biofilm structures through various mechanisms. This may include blocking the initial adhesion of microbial cells to surfaces, disrupting QS communication, and inhibiting the production of the EPM [39,40,41,42,43,44,45,46,47,48,49,50,51,52,53,54,55,56,57,58,59,60,61,62,63,64,65,66,67,68,69,70,71].

The *Calliandra surinamensis* leaf pinnulae lectin (CasuL) has been shown to inhibit biofilm formation by *S. aureus* isolates, including the non-resistant ATCC 6538 strain and the oxacillin-resistant UFPEDA-670 strain, as well as by *Staphylococcus saprophyticus*, in a dose-dependent manner. Effective concentrations of CasuL ranged from 6.25 to 800 μg/mL, depending on the strain [39]. At lower concentrations, CasuL also inhibited biofilm formation by an *S. aureus* isolate from bovine mastitis (1.78 and 3.75 μg/mL) and a *Staphylococcus* sp. isolate (Ssp5D) from caprine mastitis (0.93–3.75 μg/mL) [40].

Complementing these findings, other lectins have also exhibited antibiofilm activity, even against drug-resistant strains of *S. aureus*. Ferreira et al. [41] studied the *Alpinia purpurata* inflorescence lectin (ApuL) against *S. aureus* (UFPEDA-02), reporting a 50–60% reduction in biofilm formation at concentrations ranging from 1.56 to 50 μg/mL. The *Crataeva tapia* bark lectin (CrataBL) demonstrated biofilm inhibitory effects against various *S. aureus* strains—including methicillin-sensitive (ATCC 29213), methicillin-resistant (ATCC 33591), and vancomycin-resistant clinical isolates—at concentrations ranging from 0.18 to 0.72 μg/mL [42]. CiL-1 and CiL-2, lectins from the green alga *Codium isthmocladum*, reduced *S. aureus* biofilm biomass across all tested concentrations (15.62–500 μg/mL), with CiL-1 also reducing colony-forming unit (CFU) counts in the biofilm [43]. Similarly, the lectin BSL from the red alga *Bryothamnion seaforthii* also reduced *S. aureus* biofilm mass [44].

Further studies demonstrated that the *Punica granatum* sarcotesta lectin (PgTeL) inhibited biofilm formation by the non-resistant *S. aureus* 8325–4 strain by more than 50% at 200 μg/mL and by the methicillin-resistant LAC USA300 strain at concentrations starting from 25 μg/mL [45]. The *Myracrodruon urundeuva* heartwood lectin similarly reduced biofilm produced by 8325–4 at 50–400 μg/mL and was effective against LAC USA300 at 400 μg/mL [46]. A C-type lectin from *Bothrops jararacussu* venom exhibited a dose-dependent inhibitory effect on biofilm formation by both *S. aureus* and *S. epidermidis* at concentrations ranging from 1.56 to 100 μg/mL [47]. The *Haliclona* (*Reniera*) *implexiformis* lectin (HiL) significantly reduced the biofilm biomass of both *Staphylococcus* spp. at 500 μg/mL, also decreasing viable cell counts [48].

The *Vicia ervilia* seed lectin (VEA) demonstrated antibiofilm activity against both *S. aureus* and *S. epidermidis*. In this study, VEA was purified from six genetically distinct *V. ervilia* accessions—i.e., different landraces or varieties collected from specific Mediterranean regions (Turkey, Italy, Cyprus, and Spain). The antibiofilm activity varied depending on the accession. Specifically, VEA from accessions #5, #12, #36, and #46 inhibited biofilm formation in both bacterial species, while accession #23 was only active against *S. epidermidis*, and accession #21 showed no activity. Interestingly, all VEA samples shared identical nucleotide and amino acid sequences, except for accession #36, which presented two amino acid substitutions out of 275. Importantly, carbohydrate-binding residues were conserved across all accessions. However, these minor variations did not correlate with differences in biofilm inhibition, suggesting that the observed variability is not attributable solely to the primary structure of the protein. Instead, the authors speculate that co-purified small molecules, such as plant-derived polysaccharides, might modulate the antibiofilm effects of the lectins. Notably, accessions #5 and #36 were the most effective in reducing biofilm mass (up to 50%) in both Gram-positive strains. The authors inferred that the choice of plant accession is a critical factor in optimizing lectin-based antibiofilm strategies [49].

For instance, *Ircinia strobilina* lectin also exhibited antibiofilm activity against both *S. aureus* and *S. epidermidis*, significantly reducing total biofilm biomass and the number of viable cells [50]. The *Cratylia floribunda* (CFL) and *Vatairea macrocarpa* (VML) lectins, each at 250 μg/mL, inhibited *S. aureus* biofilm biomass formation; however, only VML was effective against *S. epidermidis* [44].

Biofilms formed by *Listeria* spp. have also been targeted by antimicrobial lectins. The water-soluble lectin from *Moringa oleifera* seeds (WSMoL) inhibited biofilm formation by *Listeria monocytogenes* strain N53-1 by 95% at 1.95 μg/mL [51]. PgTeL reduced biofilm formation by the N53-1 and EGD-e strains of *L. monocytogenes* by at least 50% at concentrations of 6.25 and 12.5 μg/mL, respectively [52]. Concanavalin A (ConA), a lectin from *Canavalia ensiformis* seeds, inhibited biofilm formation by *L. monocytogenes* serotype 4b (ATCC 19115) by 140-fold at 100 μg/mL [53]. Several fungal lectins, including *Aleuria aurantia* lectin (AAL), *Coprinopsis* galectin 2 (CGL2), *Agaricus bisporus* lectin (ABL), *Clitocybe nebularis* lectin (CNL), *Sordaria macrospora* perithecium-associated transcript (TAP1), and *Coprinopsis cinerea* mucin-binding lectin 1 (CML1), also significantly reduced the viability of *Listeria innocua* biofilms, with some also affecting *L. monocytogenes* biofilms [54].

Arfin et al. [55] reported that the *Solanum lycopersicum* fruit lectin exhibited antibiofilm activity against *Escherichia coli* at concentrations ranging from 250 to 1000 μg/mL. Similarly, lectins derived from the marine alga *Solieria filiformis*, in both native (SfL) and recombinant (rSfL-1) forms, reduced *E. coli* biofilm formation, with rSfL-1 being effective at concentrations from 3.9 to 250 μg/mL and SfL showing activity at 125 and 250 μg/mL [56]. The *Cucurbita pepo* exudate phloem lectin (CPL) inhibited *E. coli* biofilm formation by 57.06% at 200 μg/mL [57]. The *Manilkara zapota* seed lectin (MZSL) inhibited biofilm production by *E. coli* in a dose-dependent manner, with concentrations ranging from 31.25 to 500 μg/mL [58].

Uropathogenic *E. coli* strain UTI89 showed a strong response to recombinant lectin-like proteins from *Lactobacillus rhamnosus* (Llp1 and Llp2), corresponding to predicted L-type lectin domains. Llp2 inhibited biofilm formation by approximately 95% at 200 μg/mL, while Llp1 showed about 90% inhibition, with minimal effective concentrations of 10 μg/mL for Llp2 and 20 μg/mL for Llp1 [59]. In another study, biofilm formation by the enterohemorrhagic *E. coli* (EHEC) strain EDL933 was reduced 33-fold and 63-fold by ConA at concentrations of 100 and 500 μg/mL, respectively [53]. Additionally, PgTeL exhibited antibiofilm activity against *E. coli* isolates producing various β-lactamases (including CTX-M-14, CMY-2, CTX-M-14/CMY-2, CTX-M-1, and a putative metallo-β-lactamase), causing ≥50% inhibition of biofilm formation at concentrations of 6.25 μg/mL or higher [60].

The *Aplysina fulva* lectin significantly reduced the biofilm biomass of *S. aureus*, *S. epidermidis*, and *E. coli*, while also decreasing CFU counts in *E. coli* biofilms [61]. Similarly, the *Aplysina lactuca* lectin significantly reduced the biomass of *S. aureus* and *E. coli* biofilms, affecting the viability of cells within these biofilms [62].

*Pseudomonas aeruginosa* isolates have also been targeted by antibiofilm lectins. The seed lectin from *Litchi chinensis* demonstrated dose-dependent antibiofilm activity against this species, with effects observed starting at 150 μg/mL. Assessment of total protein content revealed a progressive decrease in bacterial biomass with increasing lectin concentrations [63]. *Cratylia floribunda* seed lectin (CFL) and the lectin from the red alga *Hypnea musciformis* (HML) reduced the biofilm biomass of *P. aeruginosa* at 250 μg/mL [44]. PgTeL also exhibited significant antibiofilm activity against *P. aeruginosa* strains ATCC 27853, UFPEDA 261, and UFPEDA 262, with the latter two being multidrug-resistant. PgTeL was more effective against the resistant strains, achieving 50% biofilm inhibition at minimal concentrations of 0.78 μg/mL for UFPEDA 262, 3.12 μg/mL for UFPEDA 261, and 25.0 μg/mL for the non-resistant ATCC 27853 strain [64]. A recombinant hemolymph plasma lectin (rHPL_OE_) cloned from the crab *Tachypleus tridentatus* interacted with the biofilm matrix of *P. aeruginosa* PA14, with this interaction abolished when pre-incubated with L-rhamnose [65].

The Llp1 and Llp2 lectins also showed antibiofilm activity against *Salmonella typhimurium* ATCC 14028. At 200 μg/mL, Llp1 and Llp2 reduced biofilm formation by approximately 50% and 90%, respectively. Llp2 was effective at concentrations as low as 10 μg/mL. When added after *S. typhimurium* adhered to polystyrene pegs, biofilm formation was reduced by 20% with Llp1 and 92% with Llp2, both at 50 μg/mL. Testing against ten other *Salmonella* strains showed that Llp2 consistently inhibited biofilm formation by 50–90%, whereas Llp1 was effective against only three strains [59].

Lectins have also demonstrated antibiofilm activity against a variety of other bacterial genera. CFL, BBL (*Bauhinia bauhinioides* lectin), BSL, and HML were shown to reduce the biomass of *Klebsiella oxytoca* biofilms [44]. Additionally, AB119, a bacterial lectin from *Acinetobacter baumannii*, modulated the biofilm-forming capacity of *Klebsiella pneumoniae* and *Enterococcus faecalis* [66]. The seed lectin from *Bauhinia variegata* (BVL and rBVL-1) significantly reduced the early adhesion of *Streptococcus mutans* and *Streptococcus sanguinis* to saliva-coated surfaces at 200 μg/mL, with BVL showing greater activity due to its higher proportion of dimeric forms [67]. WSMoL inhibited *Serratia marcescens* biofilm formation at concentrations ranging from 0.325 to 1.3 μg/mL. In *Bacillus* sp., WSMoL at 20.8 and 41.6 μg/mL significantly suppressed bacterial growth, preventing biofilm formation altogether [11]. Additionally, the lectin-like protein LlpA from *Burkholderia cenocepacia* exhibited antibiofilm activity against *Burkholderia ambifaria*, inhibiting biofilm formation by 52% and 36% at 5.8 μmol/L and 58 nmol/L, respectively [68].

Marine-derived lectins have also shown promising results. The hemolymph lectin from *Portunus pelagicus* (Pp-Lec) reduced biofilm formation by *Citrobacter amalonaticus*, *Vibrio parahaemolyticus*, *P. aeruginosa*, and *Proteus vulgaris* by 62–90% at 50 μg/mL [69]. Similarly, lectins from *Metapenaeus monoceros* at 50 and 100 μg/mL effectively inhibited biofilm formation by *Aeromonas hydrophila*, *V. parahaemolyticus*, *S. aureus*, and *E. faecalis* [70]. Finally, lectins from *Penaeus semisulcatus* impaired biofilm development by *V. parahaemolyticus* and *A. hydrophila* [71].

### 3.2. Lectins Can Also Eradicate Preformed Biofilms

**Figure 3:**
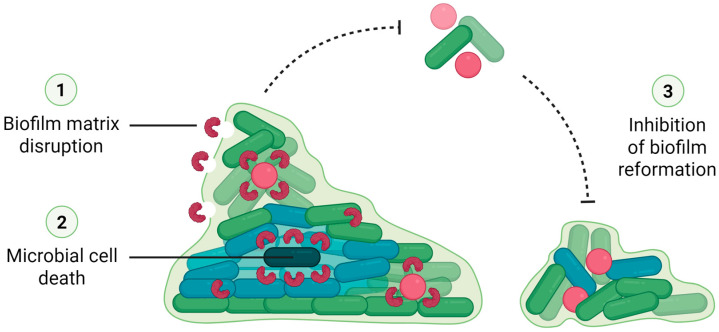
Mechanisms by which lectins disrupt pre-formed bacterial biofilms. Lectins can act on mature biofilms through distinct and complementary mechanisms: (1) matrix disruption—lectins interact with components of the extracellular polymeric matrix, leading to structural destabilization; (2) microbial cell death—lectins, after penetrating into the biofilm, may bind selectively to microbial surfaces, promoting damage or removal of target cells; (3) inhibition of biofilm reformation—lectins can prevent the dispersal of planktonic cells from contributing to the establishment of new biofilm structures. In the scheme, dashed arrows represent inhibition, indicating blockage of cell dispersal and subsequent biofilm proliferation.

In addition to inhibiting the formation of biofilms by various bacterial species and isolates, some lectins have also been reported to eradicate pre-existing biofilms by breaking down the EPM, killing or removing microbial cells, and/or preventing the biofilm from reforming (Figure 3) [11,42,47,53,54,64,65,68].

PgTeL (0.78–1.56 μg/mL) was able to eradicate biofilms formed by *P. aeruginosa* strains ATCC 27853, UFPEDA 261, and UFPEDA 262 [64]. Similarly, rHPL_OE_ at concentrations of 2.5 and 5 µM reduced preformed biofilms of *P. aeruginosa* PA14 by 16% and 24%, respectively [65].

CrataBL, both in its free form and encapsulated in liposomes, reduced the pre-existing biofilms of *S. aureus* isolates, including methicillin-sensitive, methicillin-resistant, and vancomycin-resistant strains [42]. Biofilms formed by *S. aureus* and *S. epidermidis* were also disrupted by the C-type lectin from *B. jararacussu* venom [47].

Moura et al. [11] reported that WSMoL (1.3–104 μg/mL) did not significantly reduce the biomass of pre-existing *S. marcescens* biofilms; however, scanning electron microscopy revealed hollow biofilm structures, indicating bacterial cell death. In contrast, WSMoL at concentrations of 52, 104, and 208 μg/mL effectively eradicated *Bacillus* sp. biofilms. The lectin LlpA from *B. cenocepacia* AU1054 eradicated biofilms of *B. ambifaria* LMG 19182 by reducing the number of CFU within the biofilm matrix [68].

The ability of ConA to eradicate biofilms formed by *E. coli* (EHEC) EDL933 and *L. monocytogenes* serotype 4b (ATCC 19115) was also evaluated. Only minimal biofilm dispersal was observed, suggesting a lack of significant bacterial detachment [53]. In contrast, CGL2 was found to induce the dispersal of *L. innocua* biofilms [54].

Although the antibiofilm potential of lectins has attracted increasing attention, most studies have focused on preventing biofilm formation rather than disrupting mature biofilms. This trend likely reflects the greater technical challenges involved in eradicating established biofilms, which are more resistant to antimicrobials due to their protective extracellular matrix [14,15,16,17]. Furthermore, the preventive approach aligns with the clinical interest in interfering with early bacterial adhesion, an activity in which lectins are particularly effective due to their carbohydrate-binding properties [67].

### 3.3. Dual Role of Lectins in Biofilm Modulation: Inhibition vs. Promotion

In addition to inhibiting and eradicating biofilms, studies have also shown that lectins, depending on their concentration and microbial culture conditions, can sometimes stimulate biofilm formation by bacterial isolates [40,45,51,56,59].

PgTeL, for instance, exhibited a stimulatory effect on biofilm formation by *S. aureus* 8325–4 when applied at concentrations lower than those required to impair biofilm development. The authors suggest that this increased biofilm production may represent a defensive response triggered by the detection of lectin presence at subinhibitory concentrations—levels insufficient to kill the bacteria or inhibit biofilm formation [45]. Conversely, WSMoL induced biofilm formation by *L. monocytogenes* N53-1 at concentrations ranging from 3.8 to 31.2 μg/mL, which are higher than those found to inhibit biofilm formation [51].

CasuL was found to increase biofilm production by the *Staphylococcus* sp. isolate Ssp6PD, associated with bovine mastitis [40]. SfL, in both its native and recombinant forms, increased biofilm biomass produced by *P. aeruginosa*. However, only the recombinant form of SfL resulted in a slight reduction in the number of viable bacterial cells [56]. Interestingly, Petrova et al. [59] reported that the lectins Llp1 and Llp2 enhanced biofilm formation by *Lactobacillus* strains, which are non-target and non-pathogenic bacteria.

Biofilm stimulation is not an undesirable consequence only associated with lectin exposure. It is well established that sub-inhibitory concentrations of antibiotics often act as agonists of bacterial biofilm formation. These concentrations can trigger stress responses that promote the synthesis of adhesins or EPM components, among other effects [72,73]. For instance, in *P. aeruginosa*, the aminoglycoside response regulator (*arr*) gene is involved in a defensive response to aminoglycosides that leads to changes in the levels of cyclic di-guanosine monophosphate (c-di-GMP), a QS component that regulates cell surface adhesiveness [74]. Moreover, Oliveira et al. [75] demonstrated that natural isolates of *P. aeruginosa* within biofilms produce the antibiotic pyocin, which acts as a competitive molecule that enhances the attachment of *P. aeruginosa* cells to the biofilm, while simultaneously excluding competing bacterial species.

### 3.4. Lectin-Drug Synergism and Delivery Approaches

Beyond their individual antibiofilm activity, lectins have also been studied in combination with conventional antimicrobials and incorporated into delivery systems to enhance efficacy, stability, and targeted action. In their study, Procópio et al. [40] evaluated the antibiofilm activity of a combination of CasuL and antibiotics. They observed a synergistic effect between CasuL and tetracycline against *S. aureus* isolates from bovine mastitis and *Staphylococcus* sp. (Ssp5D) from caprine mastitis.

Although still limited, some delivery strategies for antibiofilm lectins have been explored. As previously mentioned, CrataBL demonstrated antibiofilm activity against *S. aureus* strains when encapsulated in liposomes [42]. Jacalin, the jackfruit lectin, also exhibited antibiofilm properties when formulated as hydrocolloid nanoconjugates (JCuS NPs), effectively inhibiting biofilm formation and disrupting preformed biofilms of three methicillin-resistant *S. aureus* strains [76]. These findings highlight the potential of combining lectins with conventional drugs and advanced delivery systems as promising strategies to overcome biofilm-associated resistance.

### 3.5. Mechanisms of Biofilm Inhibition by Lectins

**Figure 4:**
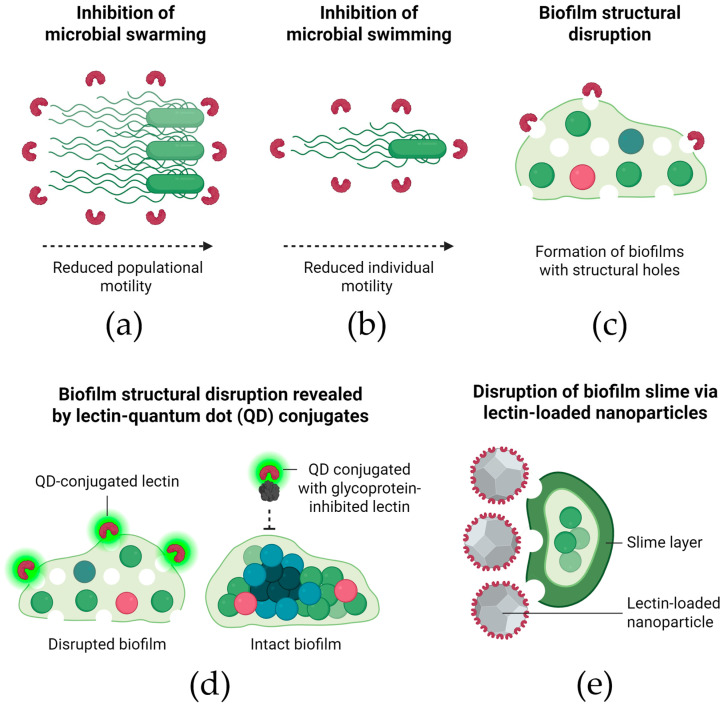
Mechanisms by which lectins inhibit the formation of new microbial biofilms. The panel illustrates distinct antibiofilm mechanisms through which lectins impair the structure and function of mature biofilms: (**a**) inhibition of microbial swarming—lectins interfere with coordinated, population-wide flagellar motility, reducing bacterial spreading capacity; (**b**) inhibition of microbial swimming—lectins impair individual bacterial motility, limiting surface colonization and biofilm expansion; (**c**) biofilm structural disruption—lectins induce structural disorganization of the biofilm matrix, generating visible holes and compromising overall cohesion; (**d**) biofilm disruption revealed by lectin–quantum dot (QD) conjugates—QD-tagged lectins localize to and disrupt biofilm regions, while glycoprotein-mediated inhibition reduces lectin binding and preserves biofilm integrity; (**e**) slime layer disruption via lectin-loaded nanoparticles—lectin-functionalized nanoparticles act directly on specific biofilm-associated components, promoting damage and weakening biofilm protection.

The antibiofilm activity of lectins has been associated with different modes-of-action (Figure 4), which can include disruption of the extracellular matrix and QS modulation, as well as impacting on bacterial motility and metabolism [11,47,53,59,63,64,65,71,76].

Effects on bacterial motility have been particularly well-documented. A reduction in the swarming motility (Figure 4a) of *P. aeruginosa* cells was associated with the inhibitory effect of *Litchi chinensis* seed lectin [63]. *Escherichia coli* EHEC cells treated with ConA exhibited reduced swimming motility (Figure 4b), which the authors considered relevant for hindering bacterial attachment and early biofilm formation [53].

The timing of lectin exposure also appears to influence their efficacy, depending on the stage of biofilm development. The antibiofilm effects of Llp1 and Llp2 from *Lactobacillus rhamnosus* against *S. typhimurium* ATCC 14028 biofilms were found to depend on the stage of the biofilm formation. Antibiofilm activity was observed when the lectins were present from the beginning of the process but was absent when they were added during the exponential growth phase. In contrast, their antibiofilm activity against *E. coli* UTI89 remained effective, even when the lectins were added during the exponential phase [59].

In addition to motility and timing, structural changes in biofilms have also been reported following lectin treatment. Petrova et al. [59] also reported that biofilms of *S. typhimurium* ATCC 14028 and *E. coli* UTI89 formed in the presence of *L. rhamnosus* lectins displayed large structural holes under microscopic observation (Figure 4c). Similarly, the typical cellular clusters and extracellular polymeric matrix of *S. aureus* and *S. epidermidis* were not observed when these bacteria were induced to form biofilms in the presence of a C-type lectin from *B. jararacussu* venom [47]. Confocal microscopy analysis revealed that a lectin from *P. semisulcatus* shrimp reduced the thickness of biofilms formed by *V. parahaemolyticus* and *A. hydrophila* [71].

These structural effects are supported by evidence of direct interaction between lectins and biofilm matrix components. The distribution of FITC-labeled Llp1 and Llp2 throughout the biofilms of *S. typhimurium* ATCC 14028 and *E. coli* UTI89 confirmed their interaction with the biofilm matrix [59]. Silva et al. [64] demonstrated, using quantum dot–PgTeL conjugates, that PgTeL caused structural damage and disruption of *P. aeruginosa* biofilms. The involvement of PgTeL’s carbohydrate recognition domains was confirmed by blocking experiments with fetuin, which prevented binding of the conjugates to the biofilms (Figure 4d).

Some lectins also interfere with bacterial communication systems, further impairing biofilm stability. The antibiofilm effect of rHPL_OE_ on *P. aeruginosa* PA14 was attributed to its binding to di-rhamnolipid, a key QS molecule. The authors further demonstrated that rhamnose-binding activity is essential to this effect, as a recombinant variant lacking this property failed to inhibit biofilm formation [65]. Similarly, mannose reduced the biofilm-inhibitory activity of ConA [53].

Other studies have linked lectin activity to alterations in membrane integrity or metabolic function. When glass surfaces were coated with WSMoL (116 μg/cm^2^), Moura et al. [11] observed that although WSMoL did not prevent *Serratia marcescens* biofilm formation, it caused membrane damage to the bacterial cells. In contrast, for *Bacillus* sp., WSMoL interfered with cell attachment to the glass surface without affecting membrane integrity. The inhibitory activity of *L. chinensis* seed lectin on *P. aeruginosa* biofilms was linked to a reduction in metabolic function, as evidenced by decreased proteolytic activity [63].

Lectin effects on specific biofilm-associated components, such as curli fibers and slime, have also been explored. Jin et al. [53] also evaluated the effect of ConA on curli fiber synthesis in EHEC biofilms but found no significant differences compared to untreated control cells. Furthermore, JCuS NPs, at their biofilm-inhibitory concentrations, inhibited slime production by *S. aureus* strains (Figure 4e). The authors emphasized that slime production is crucial for the attachment of many foodborne pathogens to food-contact surfaces [76].

Taken together, the studies reviewed in this section demonstrate that lectins act on biofilms through diverse and context-dependent mechanisms. These effects are influenced not only by the bacterial species and biofilm maturity but also by the structural and biochemical properties of each lectin. This mechanistic versatility reinforces their potential as innovative antibiofilm agents and supports ongoing efforts to tailor lectin-based strategies for specific clinical or industrial applications.

### 3.6. Challenges and Knowledge Gaps

The antibiofilm potential of lectins is broad and promising, with continued expansion anticipated. However, several challenges must be addressed—particularly the variability in strain-specific responses. For example, ApuL inhibited biofilm formation by *S. aureus* UFPEDA-02 but showed no activity against the oxacillin-resistant *S. aureus* UFPEDA-672, even at concentrations up to 400 μg/mL [41]. Similarly, the *M. urundeuva* heartwood lectin reduced biofilm production by the non-resistant *S. aureus* 8325-4 strain but was effective against the methicillin-resistant *S. aureus* LAC USA300 only at a high concentration (400 µg/mL) [46]. In contrast, PgTeL exhibited greater activity against LAC USA300 than against 8325-4 [45].

Similar strain-dependent differences were observed in other pathogens. WSMoL effectively inhibited biofilm formation by *Listeria monocytogenes* N53-1 but had no activity against the EGD-e strain [51]. Similarly, ConA, ConBol, and ConM inhibited biofilm formation by *Streptococcus mutans*, yet were ineffective against *Streptococcus oralis* [77]. These findings highlight the importance of pathogen-specific responses and suggest that lectin–biofilm interactions depend on both bacterial and lectin characteristics.

Despite the advances, several research gaps remain. Notably, there is a lack of in vivo studies and clinical data. Furthermore, there is a need for deeper investigations into the structure–function relationships of lectins, particularly in relation to their capacity to inhibit biofilm formation or disrupt established biofilms. In addition, the role of carbohydrate-binding specificity in interfering with bacterial adhesion and biofilm dynamics warrants further exploration.

Current antibiofilm strategies include the use of enzymes that degrade the extracellular polymeric matrix (EPM)—such as dispersin B, endolysins, α-amylase, and dornase alfa—as well as antibodies, nucleic-acid-binding proteins, quorum sensing (QS) inhibiting peptides, metabolic inhibitors (L-arginine, L-methionine, iron chelators), antipersister peptides, and c-di-GMP biosynthesis inhibitors (e.g., nitric oxide) [78]. Some of these agents, like lectins, are also proteins, which typically exhibit reduced stability under in vivo conditions, are costly to produce and store, and may pose immune reaction risks. On the other hand, lectins and enzymes have demonstrated efficacy in degrading the EPM and promoting biofilm dispersal, which can enhance penetration and efficacy of antibiotics [79]. Peptides, in turn, may be more effective than lectins due to their broader spectrum of action; however, this broader activity can also result in a higher level of cytotoxicity toward non-target cells [80]. In general, most antibiofilm strategies are still at the preclinical development stage [78], which is the case of lectins.

Finally, proteins, particularly in their isolated form, can exhibit varying degrees of instability, which is a critical consideration when preparing samples for both in vitro and in vivo preclinical or clinical assays. Most lectins used in antibiofilm assays are water-soluble proteins, although their solubility can vary significantly depending on their molecular structure and factors such as pH, ionic strength, temperature, and storage duration. They are typically solubilized in saline solutions (e.g., 0.15 M NaCl) [41,45], physiological buffers (e.g., PBS) [49], or water [11]. During frozen storage in aqueous solutions, lectins may precipitate due to protein aggregation, partial denaturation, mechanical damage from ice crystal formation, oxidation of amino acid residues, or the formation of unstable dimers or oligomers, among other factors [81,82]. Precipitation may be reversible or irreversible, depending on the extent of aggregation and structural damage. To improve long-term stability, lectins are often stored in lyophilized form to overcome the instability issues associated with proteins in solution [82]. However, some lectins exhibit poor resolubilization after lyophilization due to irreversible denaturation during freezing or drying, formation of insoluble aggregates, or the absence of suitable bulking agents such as cryoprotectants or lyoprotectants [82]. Thus, resolubilization efficiency depends on both the intrinsic properties of the lectin and the specific lyophilization and rehydration conditions applied. Regardless of the storage method, samples must be clarified, typically by centrifugation, prior to use in assays to remove aggregates or precipitates. Re-determining protein concentration before testing is also essential, as losses due to precipitation or surface adsorption may alter the effective concentration. Additionally, excessive or improper agitation can lead to protein denaturation or aggregation and should be carefully controlled during sample preparation.

## 4. Final Considerations

The persistent rise in antimicrobial resistance and the prevalence of biofilm-associated infections present significant challenges to global public health and medical care. Biofilms, with their complex architecture and intrinsic resistance mechanisms, protect microbial communities from both antimicrobial agents and host immune responses. As such, conventional treatments often fail to eradicate these infections, underscoring the urgent need for novel and effective antibiofilm strategies.

Lectins have emerged as promising candidates in this context, owing to their ability to selectively bind carbohydrates and interfere with key steps in biofilm development. Numerous studies have demonstrated the capacity of lectins from diverse biological sources to inhibit and, in some cases, eradicate biofilms formed by clinically relevant bacterial strains. This broad-spectrum activity highlights the versatility of lectins and their potential for application as therapeutic agents or adjuncts to existing antimicrobial treatments. Nevertheless, several challenges must be addressed before lectins can be translated into clinical use. Furthermore, understanding the molecular mechanisms underlying lectin–biofilm interactions remains a critical area for future research, which could inform the rational design of more effective and targeted antibiofilm therapies.

Overall, the evidence reviewed here reinforces the potential of lectins as innovative tools in the fight against bacterial biofilm-related infections. Continued interdisciplinary efforts are essential to unlock the full therapeutic potential of lectins and integrate them into the next generation of antimicrobial strategies.

## Figures and Tables

**Table 1:** Lectins with antibiofilm activity on bacterial isolates.

Lectins	Source	Antibiofilm Activity ^1^
**Algae**		
*Bryothamnion seaforthii* lectin (BSL)	*B. seaforthii* (red alga)	Inhibitory
*Codium isthmocladum* lectin (CiL-1 and -2)	*C. isthmocladum* (green alga)	Inhibitory
*Hypnea musciformis* lectin (HML)	*H. musciformis* (red alga)	Inhibitory
*Solieria filiformis* lectin (SfL), recombinant SfL (rSfL-1)	*Solieria filiformis* (red alga)	Inhibitory
**Animals**		
*Aplysina lactuca* lectin (ALL)	*A. lactuca* (sponge)	Inhibitory
C-type lectin	*Bothrops jararacussu* (snake) venom	Inhibitory, Eradicating
*Haliclona implexiformis* lectin (HiL)	*H.* (*Reniera*) *implexiformis* (sponge)	Inhibitory
*Ircinia strobilina* lectin (IsL)	*I. strobilina* (sponge)	Inhibitory
*Metapenaeus monoceros* lectin (MmLec)	*M. monoceros* (shrimp) hemolymph	Inhibitory
Recombinant hemolymph plasma lectin (rHPL_OE_)	*Tachypleus tridentatus* (crab) hemolymph	Inhibitory, Eradicating
Semisulcatus-lectin	*Penaeus semisulcatus* (shrimp) hemolymph	Inhibitory
*Portunus pelagicus* lectin (Pp-Lec)	*P. pelagicus* (crab) hemolymph	Inhibitory
**Microrganisms**		
*Aleuria aurantia* lectin (AAL)	*A. aurantia* (fungus)	Inhibitory
AB119	*Acinetobacter baumannii* (bacteria)	Inhibitory
*Agaricus bisporus* lectin (ABL)	*A. bisporus* (fungus)	Inhibitory
*Coprinus* galectin 2 (CGL2)	*Coprinopsis cinerea* (fungus)	Inhibitory, Eradicating
*C. cinerea* mucin-binding lectin 1 (CML1)	*C. cinerea* (fungus)	Inhibitory
*Clitocybe nebularis* lectin (CNL)	*C. nebularis* (fungus)	Inhibitory
Lectin-like protein 1 and 2 (Llp1, Llp2)	*Lactobacillus rhamnosus* (bacteria)	Inhibitory
Lectin-like protein A (LlpA)	*Burkholderia cenocepacia* (bacteria)	Inhibitory, Eradicating
Transcript associated with perithecial development 1 (TAP1)	*Sordaria macrospora* (fungus)	Inhibitory
**Plants**		
*Alpinia purpurata* lectin (ApuL)	*A. purpurata* inflorescences	Inhibitory
*Bauhinia bauhinoides* lectin (BBL)	*B. bauhinoides* seeds	Inhibitory
*Bauhinia variegata* lectin (BVL), recombinant BVL (rBVL-1)	*B. variegata* seeds	Inhibitory
*Calliandra surinamensis* lectin (CasuL)	*C. surinamensis* leaf pinnulae	Inhibitory
*Cratylia floribunda* lectin (CFL)	*C. floribunda* seeds	Inhibitory
Concanavalin A (ConA)	*Canavalia ensiformis* seeds	Inhibitory
*Cucurbita pepo* lectin (CPL)	*C. pepo* exudate phloem	Inhibitory
*Crataeva tapia* bark lectin (CrataBL)	*C. tapia* bark	Inhibitory, Eradicating
Jacalin	*Artocarpus integrifolia* seeds	Inhibitory, Eradicating
Litchi lectin	*Litchi chinensis* seeds	Inhibitory
*Myracrodruon urundeuva* heartwood lectin (MuHL)	*M. urundeuva* heartwood	Inhibitory
*Manilkara zapota* seed lectin (MZSL)	*M. zapota* seeds	Inhibitory
*Punica granatum testa lectin* (PgTeL)	*P. granatum* sarcotesta	Inhibitory, Eradicating
Tomato chitin-binding lectin (TCL)	*Solanum lycopersicum* fruit	Inhibitory
*Vicia ervilia* agglutinin (VEA)	*V. ervilia* seeds	Inhibitory
*Vatairea macrocarpa* lectin (VML)	*V. macrocarpa* seeds	Inhibitory
Water-soluble *Moringa oleifera* lectin (WSMoL)	*M. oleifera* seeds	Inhibitory, Eradicating

^1^ The type of antibiofilm effect exerted by a specific lectin—whether inhibitory or eradicating—can vary depending on the bacterial species and even among different isolates of the same species.

## Data Availability

No new data were created or analyzed in this study. Data sharing is not applicable.
